# Immunogenicity of Two FMDV Nonameric Peptides Encapsulated in Liposomes in Mice and the Protective Efficacy in Guinea Pigs

**DOI:** 10.1371/journal.pone.0068658

**Published:** 2013-07-09

**Authors:** Feng-Shan Gao, Lei Feng, Qiang Zhang, Ruo-qian Yan, Yun-Gang Li, Xin-sheng Li

**Affiliations:** 1 Department of Biochemistry and Molecular Biology, College of Life Science and Technology, Dalian University, Dalian, Liaoning, China; 2 Lanzhou Veterinary Research Institute, Chinese Academy of Agricultural Sciences, Lanzou, Gansu, China; 3 Henan Centre for Animal Diseases Control and Prevention, Animal Husbandry Bureau of Henan Province, Zhengzhou, Henan, China; 4 Shandong Center for Animal Disease Prevention and Control, Jinan, Shandong, China; 5 College of Animal Husbandry and Veterinary Science, Henan Agricultural University, Zhengzhou, Henan, China; University of California Riverside, United States of America

## Abstract

It has been predicted that nonameric peptides I (VP1_26–34_, RRQHTDVSF), II (VP1_157–165_, RTLPTSFNY) and III (VP1_45–53_, KEQVNVLDL) from the VP1 capsid protein of the foot-and-mouth disease virus (FMDV) are T cell epitopes. To investigate whether these peptides have immunological activity, BALB/c mice were immunized with peptide I, II or III conjugated with immunostimulating complexes (ISCOMs). A cytotoxic T lymphocyte assay was used to evaluate the cytotoxic activity induced by peptides along with by measuring peptide-specific T-cell proliferation and CD8^+^ T lymphocyte numbers in whole blood and interferon (IFN)-γ production in peripheral blood mononuclear cells induced by peptides. To further identify the protective efficacy of peptides, an FMDV challenge assay was done in guinea pigs. Peptides I and II stimulated significant increases in T-cell proliferation, CD8^+^ T lymphocytes, and IFN-γ secretion and cytotoxic activity compared to controls. The FMDV challenge assay indicated peptides I and II can protect over 60% of animals from virus attack. The results demonstrate that peptides I and II encapsulated in liposomes should be CTL epitopes of FMDV and can protect animals from virus attack to some extent.

## Introduction

Foot-and-mouth disease (FMD) is a highly contagious viral disease of cloven-hoofed animals that can cause great economic loss. To date, vaccination of susceptible livestock with inactivated FMD vaccine (FMDV) is the only practical means to control the epidemic in most affected countries [1]. Use of the inactivated virus as a vaccine is not completely effective because the inactivation processes are technically challenging and sometimes yield an incompletely inactivated virus. This limitation has prompted studies of safe alternative immunization strategies [2]. An epitope vaccine consisting of T- and B-cell epitopes is a safe immunogen; moreover, effective protective immunity can be induced *in vivo* if the peptide is conjugated with an appropriate adjuvant, such as complete Freund’s adjuvant (CFA) [3], lipopeptides, immunostimulating complexes (ISCOMs) or animal β_2_m fusion peptide [4]–[7]. To date, an epitope vaccine has proven to be a safe alternative that can protect animals from infection by virus [8]. The key task in designing an epitope vaccine is to identify T- and B-cell epitopes from target viruses. For FMDV, B-cell epitopes and T helper cell epitopes have been identified in the capsid protein VP1, as well as in nonstructural proteins [9]–[11]. However, B-cell and T helper epitopes mainly contribute to humoral immunity because they are restricted by major histocompatibility (MHC) class II and are dependent on CD4^+^ T lymphocyte cell activities [12]. In fact, the cellular immunity induced by the CD8^+^ cytotoxic T lymphocyte (CTL) epitope restricted by MHC class I might play a more significant role in protecting animals against FMDV [13].

In general, an epitope restricted by MHC class I is recognized by TCR and CD8 and thus induces CD8^+^ T lymphocyte activity and IFN-γ production [14]. The MHC class I-restricted epitope usually contains 8–11 amino acids that bind to the peptide-binding groove of MHC class I [15]. Two conventional methods are available for identifying MHC class I-restricted epitopes: the ^51^Cr release assay [16] and MHC–peptide tetramer staining, which is associated with a single-strand MHC class I, β_2_m, and peptides that form a tetramer complex with the CD8^+^ T lymphocyte receptor [17]. The latter method has the advantage of including the differentiation state of antigen-specific cells, allowing frequency analysis and separation of homogeneous populations of T lymphocytes. However, it should be noted that tetramer-negative cells can retain peptide-specific cytotoxicity and IFN-γ production [18]. MHC binding models have been developed for rapid and effective prediction of MHC class I-restricted epitopes using computer algorithms [19], [20]. Becker et al. used computer simulations to predict nonapeptides from FMDV OK1 and A12 strains bound to BoLA class I A11 and A20 haplotype molecules [21]. In an earlier study, we found that two nonapeptides from the VP1 capsid protein of FMDV can bind to the SLA class I complex [22], indicating that the two peptides might be CTL epitopes of FMDV. However, MHC binding does not guarantee immunogenicity [23], so a peptide immunization assay should be used in animal models. With the need for strict homogeneity in the genetic background of animals used for testing the immunological activity of peptides, such as pure inbreeding, same age and very similar body weight, the mouse has been chosen as an ideal animal model for studying the activity of FMDV epitopes [24]–[26]. In addition, earlier studies found that xenogeneic recognition of swine MHC class I molecules by mice or human TCR in CD8 molecules occurred [27], [28], indicating that mice or human CD8^+^ T lymphocytes might recognize antigenic peptides bound to pig SLA-I molecules. Until now, no MHC class I-restricted epitopes from FMDV associated with swine leukocyte antigen (SLA) class I have been identified experimentally.

We designed a mouse immunization assay and a FMDV challenge assay in geuinea pig to investigate whether the peptide has immunogenicity and can induce peptide-specific cellular immunity to protect animals from FMDV attack. The assay included measurement of peptide-specific T lymphocyte proliferation, the number of CD8^+^ cells, IFN-γ production and use of a CTL assay followed by FMDV challenge assay. We found that peptides I and II should be CTL epitopes of FMDV and can protect animals from virus attack to some extent.

## Materials and Methods

### Animals and Cell Lines

Female BALB/c mice (6 weeks old) were obtained from the Beijing Vital Laboratory Animal Technology Company (Beijing, China) and were maintained under pathogen-free conditions. Male and female guinea pigs (FMDV-free, 250–300 g body weight) were obtained from the Institute of Dalian Medical University and housed in disease-free isolation facilities. The animals were treated kindly and were used according to the animal protocols approved by the Animal Welfare and Research Ethics Committee of the Institute of Dalian Medical University (Permit Number: SYXK2010–0129). We performed the animal experiments at the Institute of Dalian Medical University. All surgery was performed under sodium pentobarbital anesthesia, and all efforts were made to minimize suffering. H22 cells, which had been established as a BALB/C mouse origin HCC cell line, were purchased from China Center for Type Culture Collection.

### Peptide Preparation

The peptides used in this study are given in Table 1. Predictions for peptides I, II and III were made using by NetMHCpan-2.0 (http://www.cbs.dtu.dk/services/NetMHCpan) [29] in combination with T-cell epitope prediction by GENETYX software (version 9.0; Software Development, Tokyo, Japan) and were synthesized by Sangon Inc. Peptide I (VP1_26–34_, RRQHTDVSF) is embedded in a B-cell epitope in positions 21–40 of the FMDV O subtype of the VP1 protein [11]. Peptide II (VP1_157–165_, RTLPTSFNY) has four overlapping amino acids within the 140–160 region of the VP1 G-H loop [10]. Peptide III (VP1_45–53_, KEQVNVLDL) is positioned in the 45–53 sites of the FMDV O subtype of the VP1 protein. In addition, two unrelated peptides (IV and V), which were from AIV H9N2 and the Grass carp reovirus, respectively, were selected at random to be used as control peptides in this study.

**Table 1:** Three synthetic peptides from the VP1 capsid protein of FMDV and two unrelated peptides from AIV H9N2 and Grass carp reovirus, respectively.

Peptide	Sequence	Position	Serotype	Protein
I	RRQHTDVSF	26–34	O	VP1
II	RTLPTSFNY	157–165	O	VP1
III	KEQVNVLDL	45–53	O	VP1
IV	KILTIYSTV	523–531	H9N2	Avian influenza virus
V	QPNEAIRSL	63–71	VP7	Grass carp reovirus

### Peptide-liposome Formation

Liposomes were manufactured by a rehydration entrapment method according to Lipford GB et al. [30] and modified. To begin with, 20.0 mg phosphatidylcholine (type XI-E), 2.0 mg phosphatidylglycerol and 10.0 mg cholesterol in a 20∶2∶10 ratio, were suspended in 5.0 ml chloroform in a 100 ml flat-bottomed flask. All the reagents were mixed by rotating the flask until the mixture was formed evenly. Next, the flask was shaken in 20 rpm in a desk-top-incubation (Infors Inc. Sweden) until all the lipid was evaporated completely and a thin lipid film formed on the flask bottom. The peptide (3.0 mg) was solved in 0.1 ml water and diluted to 1.0 ml with phosphate-buffered saline (PBS) containing 0.4 mg Quil A (Sigma, Munich, Germany). The peptide solution was slowly added to the dried lipid film and hand shaken until the lipids were resuspended. The crude peptide-liposome mixture was incubated for 30 min at room temperature, then transferred at 6000 rpm for 5 min in an Eppendorf microfuge. The mixture was then filter extrude through a 0.2 µm Anotop 10 syringe mount filter (Millipore, USA). Quality of liposome formation was assessed by electron microscopy using peptide-containing liposomes.

### Peptide Immunization

For mouse immunization, the peptide–liposomes were subcutaneously injected into mice (50 µl per animal, containing 150 µg peptides) in week 7 under control PBS. In addition, Quil A containing liposomes was used as an adjuvant control. Each group comprised 20 mice, except for the PBS and liposome control groups (*n* = 15). Each group was subdivided into groups of five mice for immunological assays. Booster immunization was carried out in week 9 and using same dose of peptide–liposomes, PBS and liposomes as the prime immunization. Samples were taken after 11, 12, 13, 14 and 15 weeks. The schedule used for mouse peptide immunization is given in Table 2.

**Table 2:** Schedule for peptide immunization.

Group	Immunogen	Dose (µl)	Groups (*n*)				Time (weeks)		
			MTT	CD8^+^	IFN-γ	CTL	Immunization	Booster	Analysis
1	PBS	50	–	5	5	5	7	9	11–15
2	Liposome	50	–	5	5	5	7	9	11–15
3	Liposome-peptide I	50	5	5	5	5	7	9	11–15
4	Liposome-peptide II	50	5	5	5	5	7	9	11–15
5	Liposome-peptide III	50	5	5	5	5	7	9	11–15
6	Liposome-peptide IV	50	5	5	5	5	7	9	11–15
7	Liposome-peptide V	50	5	5	5	5	7	9	11–15

### T-cell Proliferation

T-cell proliferation was measured using a 3-(4,5-dimethylthiazol-2-yl)-2,5-diphenyltetrazolium bromide (MTT) assay performed as previously described [31]. In brief, mice were sacrificed 14 days after booster immunization and splenocytes were collected from the spleen by scraping. The cells were suspended at a concentration of 3×10^5^–5×10^5^/ml in RPMI 1640 containing 5% (v/v) fetal calf serum. The splenocytes (100 µl) were plated in triplicate in a 96-well microtiter plate and incubated at 37°C for 24 h. The cells were stimulated for 48 h with peptide (3 µg/ml), concanavilin A (ConA; 5 µg/ml) as a positive control or BSA (2 µg/ml) as a negative control. To assess cell proliferation, MTT (Sigma) was dissolved at 5 mg/ml in PBS (pH 7.2) and 5 µl of the solution was added to each well. After incubation at 37°C for 4 h, 150 µl of DMSO was added to each well to dissolve the formazan products and the mixture was left to react in the dark at 37°C for 10 min. The absorbance at 490 nm (*A*_490_) was read and data are expressed as the stimulation index (SI) calculated as mean *A*_490_ for triplicate wells. The response was considered significant only when SI was ≥2.5.

### CD8^+^ T Lymphocyte Assay by Flow Cytometry

CD8^+^ T lymphocytes were detected by flow cytometry essentially as previously described [32] but with a slight modification. In brief, whole blood (WB) was taken from each mouse in the CD8^+^ subgroups after booster immunization at 11 weeks. WB samples were placed in tubes containing 2% (w/v) EDTA-Na_2_ as an anticoagulant and were pooled for each group for CD8^+^ T lymphocyte assays. The karyocytes in WB were adjusted to 10^6^/100 µl and then 1 µg of FITC-conjugated rat anti-CD8α monoclonal antibody (mAb; BD Pharmingen, USA) was mixed with 100 µl of WB and incubated in the dark at 37°C for 20 min. The mixture was centrifuged at 1500 rpm for 10 min, the supernatant was discarded and the precipitate was dissolved in 500 µl of PBS and subjected to flow cytometry (Epics Elite, Beckman Coulter, Fullerton, CA, USA). Two repeats were done simultaneously for each peptide during the flow cytometry.

### IFN-γ ELISA

Peripheral blood mononuclear cells (PBMCs) were isolated from mice in each IFN-γ subgroup at 11, 12, 13, 14 and 15 weeks. PBMCs were suspended in complete RPMI 1640 culture medium and stimulated with the corresponding peptides at a final concentration of 1 µg/ml. PBMCs were plated into a flat-bottomed 96-well plate at a total of 1×10^6^ cells per well and incubated for 72 h at 37°C with 5% CO_2_ in triplicate. The supernatants were harvested and assayed for IFN-γ using a mouse IFN-γ ELISA kit (BD Biosciences, San Diego, USA) according to the manufacturer’s protocol. The absorbance at 450 nm (*A*_450_) was measured with a micro ELISA reader (Bio-Rad). For each peptide, two copies of PBMCs from one mouse were treated and analyzed simultaneously.

### CTL Assay

CTL assays were performed essentially as previously described [33] but with a slight modification. Spleen T cells isolated from mice in week 11 were cultured in the presence of 20 U/ml mouse IL-2 for 7 days. The stimulated T cells were isolated and used as effector cells in a lactate dehydrogenase (LDH; Roche) cytotoxicity assay. Syngeneic H22 cells were cultured for 24 h in Roswell Park Memorial Institute medium (RPMI) 1640 supplemented with 10% (v/v) fetal bovine serum before as target cells before use. Target cells (10^6^) in RPMI complete medium were treated with 20 µg of peptide (I, II III, IV and V), incubated for 1 h at 37°C and then diluted to 2×10^5^ cells/ml and incubated overnight. The effector cells were washed with assay medium (RPMI 1640 with 1% (w/v) BSA), co-cultured at 37°C with target cells in a 96-well, round-bottom plate for 6 h. The plate was centrifuged and the supernatants were recovered and transferred to another 96-well, flat-bottom plate. A 100-µl aliquot of LDH detection mixture was added to each well and incubated at room temperature in the dark at 37°C for 30 min and *A*_490_ was then measured. Spontaneous LDH release by target and effector cells was assayed by incubating target cells in the absence of effector cells and *vice versa*. Maximum LDH release was determined by incubating target cells in assay medium containing 1% (v/v) Triton X-100. Cell-mediated cytotoxicity was calculated as:

Cytotoxicity = (Mixture of effectors and targets – Effector control/Maximum spontaneous) × 100%.

### Challenge Study for Protective Response

Guinea pigs were divided into 7 groups of 5 animals. Group 1 was injected i.m. with 200 µl of phosphate-buffered saline (PBS) as the control. Group 2 was injected with 200 µl of liposomes. Group 3 was injected with 200 µl of peptide I–liposomes (containing 600 µg of peptides). Group 4 was injected with 200 µl of peptide II–liposomes (containing 600 µg of peptides). Groups 5–7 were injected with the same dosage of peptide III-, IV- and V-liposomes, respectively, as Groups 3 and 4. Two weeks later, Groups 2–7 were injected again with the same dose of immunogen. After another two weeks, the guinea pigs were challenged with FMDV by injection i.d. of each animal with 0.2 ml of guinea pig infectious dose 100 ID_50_ of HKN/2002 in the left rear foot. After challenge, the animals were examined daily for clinical signs of FMD, including the appearance of vesicles on the mouth and feet. The observations were terminated on day 14 post challenge, when the animals were sacrificed.

### Histopathology

After the animals were sacrificed, the hepatic and cardiac tissues of the infected animals in seven groups were collected, fixed with 10% formalin, stained with hematoxylin and eosin, embedded in paraffin wax and sectioned for the observation of any structural change of cells or tissues, which were scored as: +++, total necrosis; ++, necrosis areas exceed half of total area; +, necrosis areas less than half of total areas; –, no necrosis [34].

### Statistical Analysis

All values were expressed as the mean ± SD. A one-way ANOVA (repeated measures test) was used to determine the significance of differences among the groups. Statistical significance was set at *P*<0.05.

## Results

### Peptide-specific T-cell Proliferation

**Figure 1:**
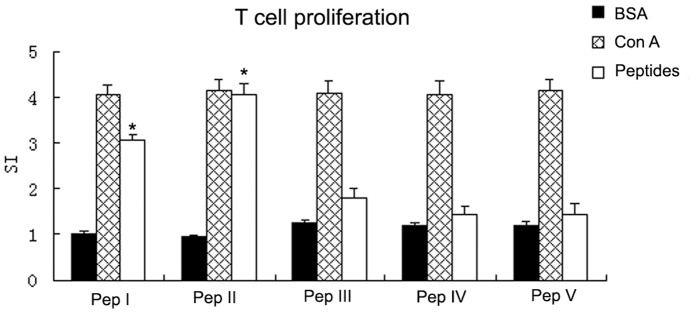
T cell activation in mice following immunization. Splenocytes were isolated from mice 2 weeks after booster immunization and were stimulated *in vitro* with peptides I–V as specific antigens. T cell proliferation was determined by the MTT method. Results are shown as the stimulation intensity (SI). *Significantly different from the BSA control group.

To determine whether peptides can induce cellular immunity against FMDV, single splenocytes were isolated from mice two weeks after booster immunization [35]. The cultures were stimulated with peptides as specific antigens; BSA was used as negative controls and ConA was used as a positive control. As shown in Figure 1, the highest levels of proliferation were observed in cultures established from mice immunized with peptide II (SI = 4.0) and peptide I (SI = 3.0). However, the SI of groups immunized with peptide III, peptide IV and peptide V was only 1.8, 1.45 and 1.45, respectively. The SI for peptides I and II was significantly different from that for the BSA control, whereas peptides III–V did not show any significant difference from the BSA control. These results suggest peptides I and II can induce a peptide-specific T-cell response.

### Detection of Specific CD8^+^ T Lymphocytes

**Figure 2:**
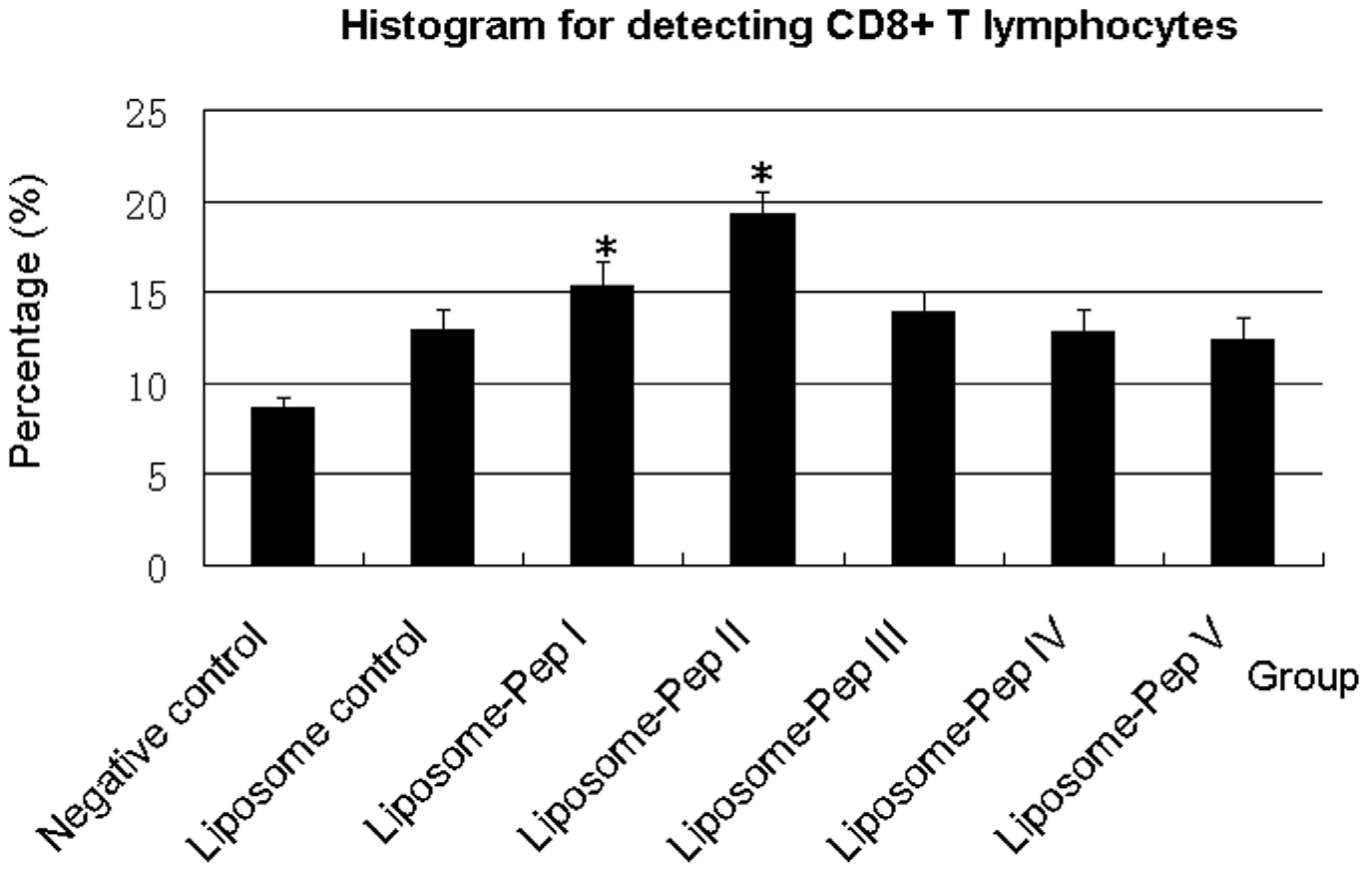
Detection of CD8^+^ T lymphocytes. Detection of CD8^+^ T lymphocytes was done after booster immunization at 11 weeks. Negative control showing only 8.7% CD8^+^ T lymphocytes in total karyoctes. Liposome control showing 12.9% CD8^+^ T lymphocytes. Peptide I showing 15.4% CD8^+^ T lymphocytes. Peptide II with 19.3% CD8^+^ T lymphocytes. Peptide III with 13.9% CD8^+^ T lymphocytes. Peptide IV with 12.8% CD8^+^ T lymphocytes. Peptide V with 12.4% CD8^+^ T lymphocytes. CD8^+^ T cell proliferation in cells stimulated with peptide I and peptide II was significantly higher than that in cells stimulated with liposomes (*P*<0.05). Cumulative average (*n* = 5) per group for the groups described in Table 2. *P<0.05 vs. Liposome control.

The generation of peptide-specific CD8^+^ T lymphocytes *in vivo* is indicative of effective cellular immunity. The peptide-specific CD8^+^ T cell clones responding in a proliferative assay were found to be class I MHC-restricted [36]. To determine whether peptides could induce CD8^+^ T cell clones to proliferate, whole blood (WB) was taken from each mouse in the CD8^+^ subgroups after booster immunization at 11 weeks. CD8^+^ T lymphocytes were detected using a rat anti-mouse CD8α mAb by flow cytometry. Mice treated with peptide I or peptide II had 15.4% and 19.3% CD8^+^ T lymphocytes, respectively, in total karyoctes (Figure 2). Results for the PBS control, the liposome control, and peptides III, IV and V were 8.7%, 12.9%, 13.9%, 12.8% and 12.4%, respectively. Peptides I and II induced significant CD8^+^ T lymphocyte increases of 2.5% and 6.4%, respectively, compared to the liposome control, whereas an increase of only 1.0% was observed for peptide III. Peptides IV and V did not induce any increase in CD8^+^ T lymphocytes compared to the liposome control. Therefore, peptides I and II were effective at inducing CD8^+^ T lymphocytes.

### Detection of Peptide-specific IFN-γ

**Figure 3:**
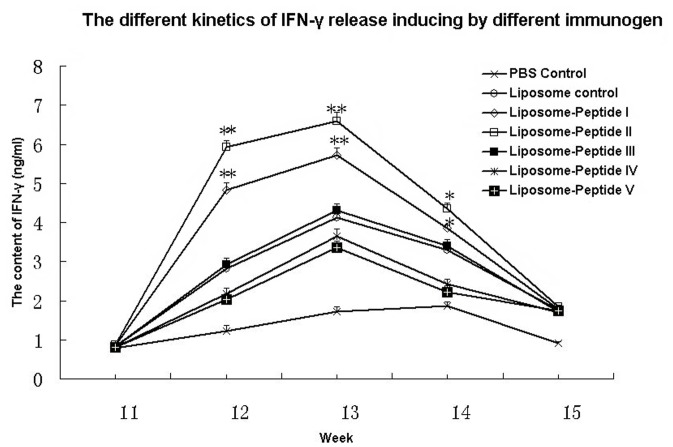
ELISA results for IFN-γ release in PBMCs from different groups of mice. IFN-γ release in PBMCs stimulated with peptides from different groups of mice was detected after boost immunization at 11, 12, 13, 14 and 15 weeks. Peptides I and II induced a significant increase in IFN-γ from 12 to 14 weeks after booster immunization compared to the liposome control, whereas peptide III showed no any significance in IFN-γ release increase compared to mice in liposome control group from 11 to 15 weeks. Unrelated peptide IV and peptide V showed a lower IFN-γ release level compared to liposome control group from 11 to 15 weeks. **P<0.01, *P<0.05 vs. Liposome control.

Previous studies demonstrated that MHC class I epitopes can induce IFN-γ release by CD8^+^ T lymphocytes [14], [37]. ELISA showed that peptides I and II induced a significant increase in IFN-γ from 12 to 14 weeks after booster immunization compared to the liposome control, whereas peptides III, IV and V had little effect (Figure 3). The results indicate that peptides I and II were effective in inducing IFN-γ production *in vivo*.

### Detection of Cytotoxicity of T lymphocytes Induced by Peptides

**Figure 4:**
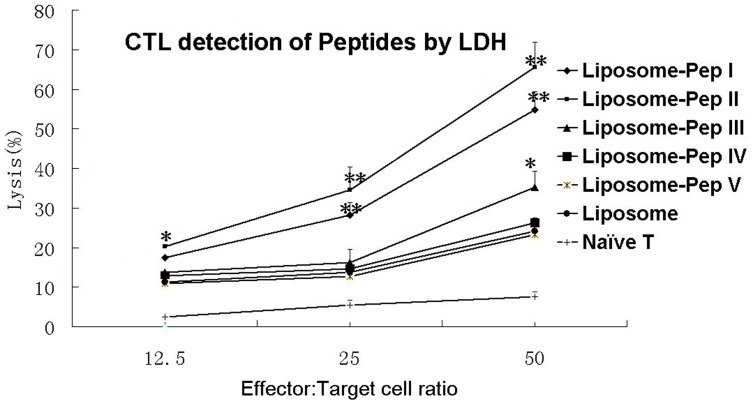
Detection of peptide cytotoxicity by LDH. Spleen T cells isolated and collected from five mice per group that had been injected with peptides, liposome or PBS after boost immunization in week 11 were induced *in vitro* for 7 days with the corresponding peptides. T cells derived from the peptide I and peptide II groups had significantly higher cytotoxic activity compared to liposome. Peptide III and unrelated peptide IV-V did not show any significant difference in cytotoxic activity compared to the liposome. **P<0.01, *P<0.05 vs. control Liposome mice.

CTL results for the peptides I and II groups were significantly different from those for the liposome (*P*<0.01) and naïve T cell groups (*P*<0.01), whereas those for unrelated peptides IV and V were not (Figure 4). Although peptide III had a significant effect at a 50∶1 effector/target cell ratio (*P*<0.05), it would be weak in inducing CTL activity for CD8^+^ lymphocytes. The results demonstrate that T cells derived from the peptides I and II groups possessed greater cytotoxic activity than those derived from peptide III and unrelated peptide groups.

### Protective Efficacy in Guinea Pig

From day 3 post challenge, animals in the PBS control group developed acute signs of FMDV, including vesicles and fever followed by animals in other groups. Guinea pigs in the Liposomes group and the unrelated peptides IV and V groups also developed clinical symptoms of FMDV from day 3 and all animals in the above groups showed signs of FMDV infection by day 7. However, peptides I, II and III can protect animals from FMDV attack to different extents. The protective assay for guinea pigs after FMDV challenge is given in Table 3. Peptide III protected 25% of animals against FMDV challenge, peptide I protected 60% of animals and peptide II protected 80% of animals. By contrast, peptides IV and V did not protect any animal.

**Table 3:** Protective efficacy for immunized guinea pigs after FMDV challenge.

Group	Numbers of guinea pigs with FMDV clinical signs post challenge (total 5 animals every group)						Protection
	Day 3–4	Day 5–6	Day 7–8	Day 9–10	Day 11–12	Day 13–14	
PBS	5	5	5	5	5	5	0
Liposome	3	4	5	5	5	5	0
Liposome-peptide I	0	0	1	1	2	2	60%
Liposome-peptide II	0	0	0	1	1	1	80%
Liposome-peptide III	0	1	2	3	4	4	25%
Liposome-peptide IV	3	4	5	5	5	5	0%
Liposome-peptide V	4	4	5	5	5	5	0%

### Histopathology

**Figure 5:**
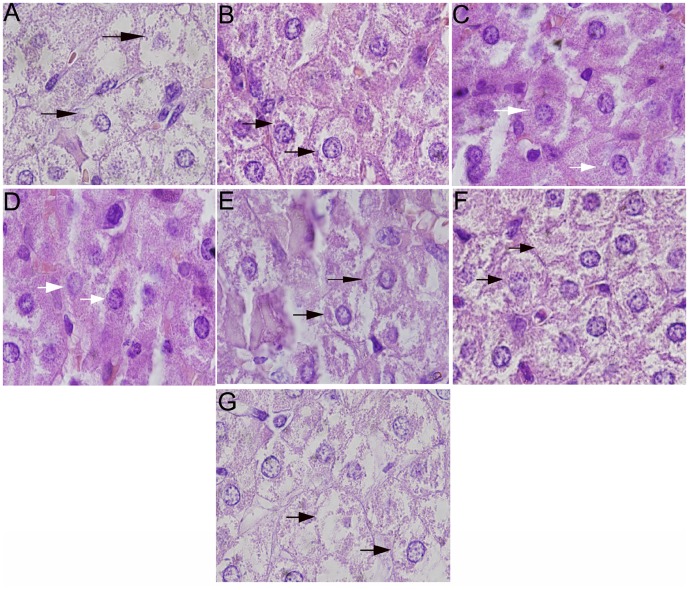
Histopathology of group animals after FMDV challenge. The sections of hepatic tissues from seven groups of guinea pigs were made after animals were sacrificed on day 14 post challenge. The hepatic histological changes in sections of different groups of animals were observed under optical microscope (40×). The black arrows pointed to the typical injured hepatic cells and the white arrows pointed to the typical uninjured hepatic cells. (A) The hepatic histological changes of guinea pigs in PBS group after FMDV challenge. The hepatic cells became swollen and vesicular without well-proportioned cytoplasm after granular degeneration in cytoplasm, and even parts of hepatic cells became vacuoles. (B) The hepatic histological changes of guinea pigs in liposome group after FMDV challenge. The hepatic cells became swollen and parts of hepatic cells developed granular degeneration in cytoplasm. (C) The hepatic histological changes of guinea pigs in peptide I group after FMDV challenge. The hepatic cells aligned regularly with slight inflammatory cell infiltration. (D) The hepatic histological changes of guinea pigs in peptide II group after FMDV challenge. The hepatic cells aligned regularly with slight inflammatory cell infiltration. (E) The hepatic histological changes of guinea pigs in peptide III group after FMDV challenge. The hepatic cells displayed swollen and some of cells developed granular degeneration in cytoplasm. (F) The hepatic histological changes of guinea pigs in peptide IV group after FMDV challenge. Most of hepatic cells became swollen with granular degeneration in cytoplasm. (G) The hepatic histological changes of guinea pigs in peptide V group after FMDV challenge. After granular degeneration in cytoplasm, the hepatic cells became vesicular, and even parts of hepatic cells became vacuoles.

Table 4 and Figure 5 show the histological changes observed after treatment. Hepatic cells in sections of the groups treated with peptides I and II were aligned regularly with slight inflammatory cell infiltration and cardiac cells were also displayed regularly without obvious cellular lesions. However, hepatic cells in the groups treated with peptides III–V group were swollen and displayed degeneration, necrosis and inflammatory cell infiltration that was more severe compared to groups treated with peptides I and II. However, cardiac lesions were not obvious, except some cardiac fibers were cracked in the PBS control group of guinea pigs. Liver damage was more severe in the PBS control group compared to the other groups.

**Table 4:** Histological changes in seven groups.

Group	Animal numbers	Numbers of animals with score of histological of cellular lesions (hepatic cells)			
		+	++	+++	−
PBS	5	0	0	5	0
Liposome	5	1	2	2	0
Liposome-peptide I	5	1	0	0	4
Liposome-peptide II	5	1	0	0	4
Liposome-peptide III	5	3	1	0	1
Liposome-peptide IV	5	1	2	2	0
Liposome-peptide V	5	0	1	4	0

## Discussion

Previous studies on FMDV epitopes mainly concentrated on B-cell epitopes and T cell helper epitopes that induced humoral immunity, which provides crucial protection against FMDV for cloven-hoofed animals [38], [39]. Recent studies indicate that cellular immunity plays an important role in preventing FMDV invasion in animals [40], especially during persistent FMDV infection [2]. Therefore, T-cell epitopes that could induce cytotoxicity against FMDV should be systematically studied. However, no MHC class I-restricted epitope from FMDV associated with swine leukocyte antigen (SLA) class I has been identified. In this research, we investigated two CTL epitopes derived from FMDV.

Detection of peptide-specific T-cell proliferation demonstrated that peptides I and II had significantly higher SI than the BSA control, whereas peptide III and the unrelated peptides did not. This confirms that peptides I and II are T-cell epitopes for FMDV. We detected CD8^+^ T lymphocytes by flow cytometry using a rat anti-mouse CD8 mAb. Mice in the peptide I and II groups had greater numbers of CD8^+^ T lymphocytes compared to the negative control, the Liposome control and peptides III, IV and V (Figure 2). Although CD8^+^ results were not obtained for single mice in each group for statistical analysis, the mean CD8^+^ group results for pooled WB indicate that peptides I and II were effective in stimulating proliferation of CD8^+^ T cells. It is noteworthy that peptide II provided the greatest stimulatory effect on CD8^+^ T-cell proliferation. IFN-γ ELISA results showed that peptides I and II, but not peptide III and the unrelated peptides, induced a significant increase in IFN-γ from 12 to 14 weeks compared to the liposome and PBS control groups. The CTL assay proved that T cells derived from peptide I and II groups had significantly higher cytotoxic activity compared to liposome (*P*<0.05) and naïve T-cell groups (*P*<0.01), but those from peptide III and unrelated peptide groups did not. The above results all suggest that peptide I and peptide II should be CTL epitopes of FMDV. Although the designed peptides were derived from FMDV serotype O, we found that peptide I (FMDV/VP1-28-34) matched at least 83 strains of FMDV serotype O and 7 strains of FMDV serotype A, while peptide II (FMDV/VP1-157-165) matched at least 89 strains of FMDV serotype O and one strain of FMDV serotype Asia1 (See Table S1), using blast in the GenBank Database. Therefore, peptide I and peptide II are suitable for multi-stains and the multi-serotypes of FMDV, which might be used to design a new FMDV epitope vaccine to protect animals.

The protected assay in guinea pigs by challenging FMDV was used to identify the peptides’ immunological activity. In this assay, peptide I and peptide II encapsulated in liposomes could protect 60 and 80 percent of group animals from FMDV challenging, respectively, as it was shown in Table 3. In contrast to other peptides group and control groups, peptides I and II for guinea pigs should have significant protection against FMDV infection, although the protection is not 100%. The histological sections also proved that peptides I and II could protect group animals from FMDV infection in tissue cells, while peptide III only can protect part of tissue cells from FMDV infection. Peptides IV, V, liposome and PBS can not protect any tissue cells from FMDV infection, as it was shown in Table 4 and Figure 5. In consideration of only peptide I or peptide II could not protect 100 percent of animal from virus infection, multi-CTL epitopes in tandem combination as DNA vaccine to immunize natural host of FMDV might be a more bright originality.

Earlier studies also identified that ISCOMs, are stable particulate complexes of protein antigens incorporated into cage-like structures consisting of the adjuvant Quil A and lipid [41], [42]. It was reported that synthetic peptides covalently linked to lipids packaged within ISCOMs could induce strong CTL responses. In this assay, we prepared ISCOMs conjugated with peptides and then they were used to immune mice. Meantime, we also used complete Freund’s adjuvant (CFA)/incomplete Freund’s adjuvant (IFA) as adjuvants to conjugate with peptides to immune mice. The results identified that the ISCOMs can induce more strong cellular immunity responses than the CFA/IFA conjugated with peptides (see Figure S1). In addition, we noticed liposome was more effective compared to the negative control group in inducing CD8^+^ T lymphocyte proliferation, IFN-γ release by different immunogens and cytotoxic T lymphocyte activity. The increased level of liposome control observed with respect to the negative control was higher than that observed between the liposome control and immunized animals with peptides I and III. These findings should explain how ISCOMs containing liposomes can induce strong cellular immunity responses. The ability of ISCOMs to stimulate cellular immunity may be relevant to design of future anti-viral epitope-vaccines.

### Conclusions

In conclusion, we demonstrated that peptides I and II from FMDV are CTL epitopes that induce peptide-specific cellular immunity in model animal of mice and protect guinea pigs from FMDV infection at some extent. These peptides might be useful for further study related to design of an FMDV vaccine.

## Supporting Information

Figure S1 **Comparing the cellular immunity responses inducing by liposomes and IFA.** Mice in IFA control group were immunized with 100 µl complete Freund’s adjuvant (CFA) and booster immunized with 100 µl incomplete Freund’s adjuvant (IFA) two weeks after. Mice in liposome group were immunized with 50 µl Quil A-containing liposomes and boosted with same dosages two weeks after. Mice in peptide II group were immunized with 150 µg peptide II conjugated with CFA and booster immunized with 150 µg peptide II conjugated with IFA two weeks after. Mice in liposome-peptide II group were immunized with 50 µl ISCOMs (liposome- peptide II) containing 150 µg peptide II and boosted with same dosages two weeks after. There were 5 mice in every group. **A**, Comparing the CD8^+^ T lymphocytes proliferation inducing by liposomes and IFA; B, comparing the IFN-γ release inducing by liposomes and IFA; C, comparing cytotoxity inducing by liposomes and IFA.
(DOC)

Table S1 **Searching FMDV/VP1-28-34 and FMDV/VP1-157-165 in Protein databank(PDB) by blast.**
(DOC)
