# Supplementary material for: Immunogenicity of Two FMDV Nonameric Peptides Encapsulated in Liposomes in Mice and the Protective Efficacy in Guinea Pigs
Source: PLoS One. 2013 Jul 9;8(7):e68658. doi: 10.1371/journal.pone.0068658 (PMC3706604; doi:10.1371/journal.pone.0068658)
Supplement: Figure S1 — Comparing the cellular immunity responses inducing by liposomes and IFA. Mice in IFA control group were immunized with 100 µl complete Freund’s adjuvant (CFA) and booster immunized with 100 µl incomplete Freund’s adjuvant (IFA) two weeks after. Mice in liposome group were immunized with 50 µl Quil A-containing liposomes and boosted with same dosages two weeks after. Mice in peptide II group were immunized with 150 µg peptide II conjugated with CFA and booster immunized with 150 µg peptide II conjugated with IFA two weeks after. Mice in liposome-peptide II group were immunized with 50 µl ISCOMs (liposome- peptide II) containing 150 µg peptide II and boosted with same dosages two weeks after. There were 5 mice in every group. A, Comparing the CD8+ T lymphocytes proliferation inducing by liposomes and IFA; B, comparing the IFN-γ release inducing by liposomes and IFA; C, comparing cytotoxity inducing by liposomes and IFA. (DOC) [file pone.0068658.s001.doc]

**A**


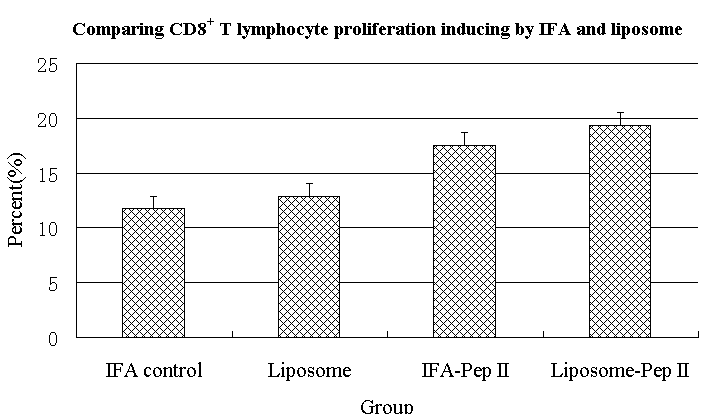


**B**


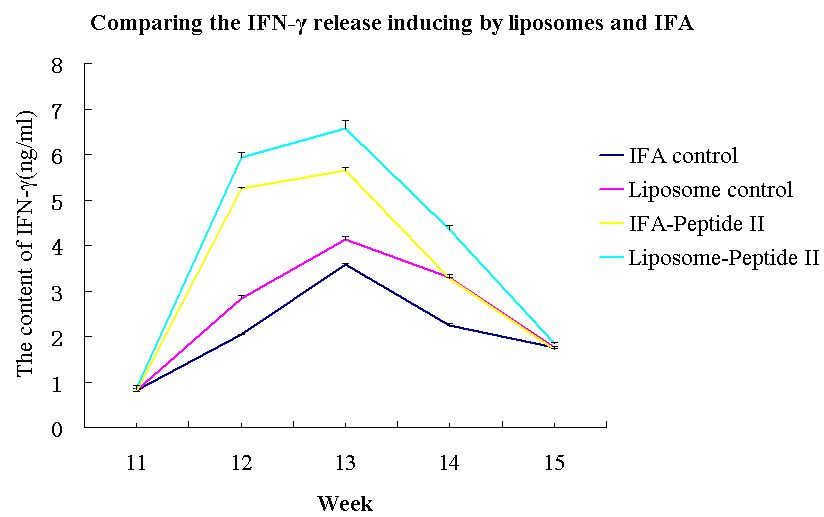


**C**


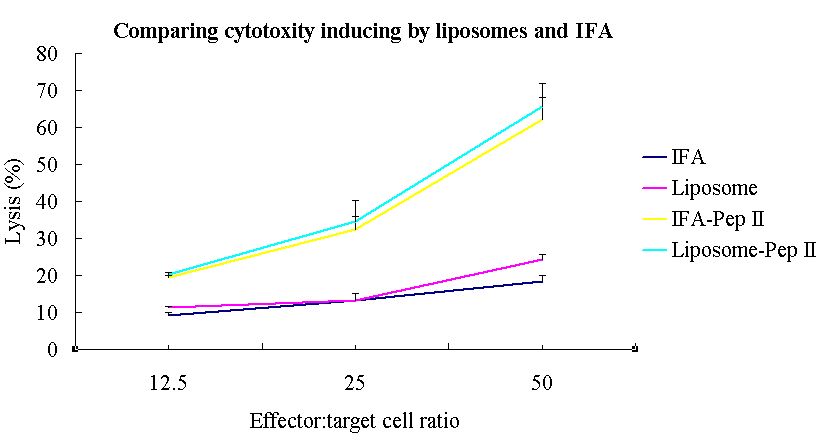


**Figure S1. Comparing the cellular immunity responses inducing by liposomes and IFA.**

Mice in IFA control group were immunized with 100 μl complete Freund’s adjuvant (CFA) and booster immunized with 100 μl incomplete Freund’s adjuvant (IFA) two weeks after. Mice in liposome group were immunized with 50 μl Quil A-containing liposomes and boosted with same dosages two weeks after. Mice in peptide II group were immunized with 150 μg peptide II conjugated with CFA and booster immunized with 150 μg peptide II conjugated with IFA two weeks after. Mice in liposome-peptide II group were immunized with 50 μl ISCOMs (liposome- peptide II) containing 150 μg peptide II and boosted with same dosages two weeks after. There were 5 mice in every group. **A**, Comparing the CD8^+^ T lymphocytes proliferation inducing by liposomes and IFA; B, comparing the IFN-γ release inducing by liposomes and IFA; C, comparing cytotoxity inducing by liposomes and IFA.
