# Supplementary material for: Immunogenicity of Two FMDV Nonameric Peptides Encapsulated in Liposomes in Mice and the Protective Efficacy in Guinea Pigs
Source: PLoS One. 2013 Jul 9;8(7):e68658. doi: 10.1371/journal.pone.0068658 (PMC3706604; doi:10.1371/journal.pone.0068658)
Supplement: Table S1 — Searching FMDV/VP1-28-34 and FMDV/VP1-157-165 in Protein databank(PDB) by blast. (DOC) [file pone.0068658.s002.doc]

**Table S1** Searching FMDV/VP1-28-34 and FMDV/VP1-157-165 in Protein databank(PDB) by blast

| Number | Genbank | Strains | Type | Country | Release year |
| --- | --- | --- | --- | --- | --- |
| *FMDV/VP1-28-34 (RRQHTDVSF) | | | | | |
| 1 | ADN03401 | SonLa-2/VIT/06 | O | Viet Nam: Son La | 2010 |
| 2 | ACD44900.1 | O/LAO/24/2003 | O | United Kingdom | 2009 |
| 3 | AAW82071 | Type A | A | Iran: Tehran | 2005 |
| 4 | CAC22198 | ETH/8/94 | O | Ethiopia | 2002 |
| 5 | ABB92798 | O/Kirsehir/TUR/172/03/03 | O | Turkey | 2006 |
| 6 | ACD44899 | O/LAO/22/2003 | O | Laos | 2009 |
| 7 | ADP24183 | Kalkilia-99 | O | Israel | 2010 |
| 8 | AAQ72777 | Type A | A | Iran | 2003 |
| 9 | CAC22168 | TUR/1/96 | O | Turkey | 2002 |
| 10 | ADP24161 | Zarzir-04 | O | Israel | 2010 |
| 11 | ABG77558 | Type A | A | Iran | 2006 |
| 12 | AEE99089 | O/Santander/Col/94 | O | Colombia | 2011 |
| 13 | ADV38199 | O/VIT/7/2002 | O | Viet Nam | 2011 |
| 14 | AAM64012 | A/IND/302/88 | A | India: Rajasthan | 2002 |
| 15 | CCF23100 | PakMulAliPur/2011 | O | PakMulAliPur/2011 | 2012 |
| 16 | AFO67901 | OGOA12002 | O | India | 2012 |
| 17 | ACN79665 | A/SAU/41/91 | A | Saudi Arabia | 2009 |
| 18 | ABP82759 | A/IRN/2/87 | A | Iran: Mardabad, Kardaj, Tehran | 2007 |
| 19 | CAC22165 | TAN/7/98 | O | Tanzania | 2002 |
| 20 | AFU81779 | B08 | O | China | 2012 |
|  |  |  |  |  |  |
| **FMDV/VP1-157-165 (RTLPTSFNY) | | | | | |
| 1 | CAC22217 | ISR/1/96 | O | Israel | 2002 |
| 2 | AAL05237 | O/IND469/98 | O | India: Uttar Pradesh | 2001 |
| 3 | AAL05203 | AAL05203 | O | India: Gujarat | 2001 |
| 9 | AAM69488 | O/IND/307/98 | O | India: Assam | 2002 |
| 4 | CAC22224 | KEN/4/95 (K29/95) | O | Kenya | 2002 |
| 4 | AAK62022 | O/IND/7/95 | O | India: Karnataka | 2002 |
| 5 | AAK62010 | O/IND/27/95 | O | India: Tamil Nadu | 2001 |
| 6 | AAK62025 | O/IND/8/92 | O | India: Assam | 2001 |
| 7 | AAK62019 | O/IND/57/96 | O | India: Andhra Pradesh | 2001 |
| 8 | AAK62005 | O/IND/17/96 | O | India: West Bengal | 2001 |
| 9 | AAK62011 | O/IND/2/96 | O | India: West Bengal | 2001 |
| 10 | AAK62021 | O/IND/6/90 | O | India: Punjab | 2001 |
| 11 | AAR85344 | asia1/wbud/26/95 | Asian I | India: West Bengal, Uttar Dinajpur | 2004 |
| 12 | AAM69482 | O/IND/51/98 | O | India: Haryana | 2002 |
| 13 | CAC22226 | KUW/1/96 | O | Kuwait | 2002 |
| 14 | CAC40794 | O Bariloche/Arg/94 | O | Argentina:Rio Negro | 2006 |
| 15 | CAC22171 | YEM/4/95 | O | Yemen | 2002 |
| 16 | CAC22194 | BAN/3/96 | O | Bangladesh | 2002 |
| 17 | CAC22202 | GRE/27/96 | O | Greece | 2002 |
| 18 | AAP81678 | CAR/16/00 | O | Cameroon | 2004 |
| 19 | CAC22231 | NEP/46/95 | O | Nepal | 2002 |
| 20 | AAR85363 | UGA/42/75 | O | Uganda | 2006 |
| 21 | AAG27037 | SAR/12/00 | O | South Africa | 2007 |
| 22 | AAR07960 | UGA/1/03/Jinja | O | Uganda | 2006 |

Note: * indicated that 14 of 83 strains of O serotypes and 6 of 7 strains of A serotype of FMDV are shown in this table. **indicated that 21 of 89 strains of O serotype and one strain of Asia1 serotype of FMDV are show in this table.
